# Supplementary material for: Resolving the full spectrum of human genome variation using Linked-Reads
Source: Genome Res. 2019 Apr;29(4):635–45. doi: 10.1101/gr.234443.118 (PMC6442396; doi:10.1101/gr.234443.118)
Supplement: Supplemental Material [file supp_gr.234443.118_Supplemental_longranger-2.2.zip › longranger-2.2.tar.bz2!/lr2.2-public/lib/stan/cmdstan/stan/doc/api/html/agrad_2rev_2matrix_2_l_d_l_t__factor_8hpp_source.html]

Stan: src/stan/agrad/rev/matrix/LDLT\_factor.hpp Source File


|  |  |
| --- | --- |
|  | Stan  2.5.0  probability, sampling & optimization |


- Main Page
- Related Pages
- Namespaces
- Classes
- Files

- File List
- File Members

All Classes Namespaces Files Functions Variables Typedefs Enumerations Enumerator Friends Macros Pages

- src
- stan
- agrad
- rev
- matrix

LDLT\_factor.hpp

Go to the documentation of this file.

1 #ifndef STAN\_\_AGRAD\_\_REV\_\_MATRIX\_\_LDLT\_FACTOR\_HPP

2 #define STAN\_\_AGRAD\_\_REV\_\_MATRIX\_\_LDLT\_FACTOR\_HPP

3

4 #include <stan/agrad/rev/var.hpp>

5 #include <stan/agrad/rev/matrix/LDLT\_alloc.hpp>

6 #include <stan/math/matrix/LDLT\_factor.hpp>

7 #include <stan/math/error\_handling/matrix/check\_square.hpp>

8

9 namespace stan {

10  namespace math {

44  template<int R, int C>

45  class LDLT\_factor<stan::agrad::var,R,C> {

46  public:

52  LDLT\_factor() : \_alloc(new stan::agrad::LDLT\_alloc<R,C>()) {}

53

54  LDLT\_factor(const Eigen::Matrix<stan::agrad::var,R,C> &A)

55  : \_alloc(new stan::agrad::LDLT\_alloc<R,C>()) {

56  compute(A);

57  }

58

67  inline void compute(const Eigen::Matrix<stan::agrad::var,R,C> &A) {

68  stan::math::check\_square("comute(%1%)",A,"A",(double\*)0);

69  \_alloc->compute(A);

70  }

71

83  template<typename Rhs>

84  inline const Eigen::internal::solve\_retval<Eigen::LDLT< Eigen::Matrix<double,R,C> >, Rhs>

85  solve(const Eigen::MatrixBase<Rhs>& b) const {

86  return \_alloc->\_ldlt.solve(b);

87  }

88

94  inline bool success() const {

95  bool ret;

96  ret = \_alloc->N\_ != 0;

97  ret = ret && \_alloc->\_ldlt.info() == Eigen::Success;

98  ret = ret && \_alloc->\_ldlt.isPositive();

99  ret = ret && (\_alloc->\_ldlt.vectorD().array() > 0).all();

100  return ret;

101  }

102

110  inline Eigen::VectorXd vectorD() const {

111  return \_alloc->\_ldlt.vectorD();

112  }

113

114  inline size\_t rows() const { return \_alloc->N\_; }

115  inline size\_t cols() const { return \_alloc->N\_; }

116

117  typedef size\_t size\_type;

118  typedef stan::agrad::var value\_type;

119

128  stan::agrad::LDLT\_alloc<R,C> \*\_alloc;

129  };

130  }

131 }

132 #endif

stan::math::LDLT\_factor< stan::agrad::var, R, C >::vectorD

Eigen::VectorXd vectorD() const

The entries of the diagonal matrix D.

**Definition:** LDLT\_factor.hpp:110

\_alloc

log\_determinant\_spd\_alloc< R, C > \* \_alloc

**Definition:** log\_determinant\_spd.hpp:30

stan::agrad::LDLT\_alloc

This object stores the actual (double typed) LDLT factorization of an Eigen::Matrix<var> along with poi...

**Definition:** LDLT\_alloc.hpp:20

LDLT\_alloc.hpp

stan::math::LDLT\_factor< stan::agrad::var, R, C >::rows

size\_t rows() const

**Definition:** LDLT\_factor.hpp:114

stan::math::LDLT\_factor< stan::agrad::var, R, C >::success

bool success() const

Determine whether the most recent factorization succeeded.

**Definition:** LDLT\_factor.hpp:94

stan::math::LDLT\_factor< stan::agrad::var, R, C >::\_alloc

stan::agrad::LDLT\_alloc< R, C > \* \_alloc

The LDLT\_alloc object actually contains the factorization but is derived from the chainable\_alloc cla...

**Definition:** LDLT\_factor.hpp:128

stan::math::LDLT\_factor< stan::agrad::var, R, C >::compute

void compute(const Eigen::Matrix< stan::agrad::var, R, C > &A)

Use the LDLT\_factor object to factorize a new matrix.

**Definition:** LDLT\_factor.hpp:67

stan::math::LDLT\_factor

**Definition:** LDLT\_factor.hpp:16

stan::math::LDLT\_factor< stan::agrad::var, R, C >::size\_type

size\_t size\_type

**Definition:** LDLT\_factor.hpp:117

var.hpp

stan::math::LDLT\_factor< stan::agrad::var, R, C >::solve

const Eigen::internal::solve\_retval< Eigen::LDLT< Eigen::Matrix< double, R, C > >, Rhs > solve(const Eigen::MatrixBase< Rhs > &b) const

Compute the actual numerical result of inv(A)\*b.

**Definition:** LDLT\_factor.hpp:85

stan::agrad::var

Independent (input) and dependent (output) variables for gradients.

**Definition:** var.hpp:27

stan::math::LDLT\_factor< stan::agrad::var, R, C >::value\_type

stan::agrad::var value\_type

**Definition:** LDLT\_factor.hpp:118

stan::math::LDLT\_factor< stan::agrad::var, R, C >::LDLT\_factor

LDLT\_factor()

Default constructor.

**Definition:** LDLT\_factor.hpp:52

stan::math::LDLT\_factor< stan::agrad::var, R, C >::LDLT\_factor

LDLT\_factor(const Eigen::Matrix< stan::agrad::var, R, C > &A)

**Definition:** LDLT\_factor.hpp:54

stan::math::LDLT\_factor< stan::agrad::var, R, C >::cols

size\_t cols() const

**Definition:** LDLT\_factor.hpp:115

stan::math::check\_square

bool check\_square(const char \*function, const Eigen::Matrix< T\_y, Eigen::Dynamic, Eigen::Dynamic > &y, const char \*name, T\_result \*result)

Return true if the specified matrix is square.

**Definition:** check\_square.hpp:24

LDLT\_factor.hpp

check\_square.hpp


---

[ Stan Home Page ]

*© 2011–2014,
Stan Development Team.*
